# Three‐dimensional computerized tomography reconstruction‐based morphologic assessment of the coracoid process in an Asian population: Clinical implications for shoulder surgery

**DOI:** 10.1002/jeo2.70109

**Published:** 2024-12-15

**Authors:** Tongyue Ji, Su Yan, Chao Lu, Hao Shu, Luning Sun

**Affiliations:** ^1^ The First Clinical Medical College Nanjing University of Chinese Medicine Nanjing China; ^2^ Department of Orthopedics Jiangsu Province Hospital of Chinese Medicine, Affiliated Hospital of Nanjing University of Chinese Medicine Nanjing China; ^3^ Department of Radiology Jiangsu Province Hospital of Chinese Medicine, Affiliated Hospital of Nanjing University of Chinese Medicine Nanjing China

**Keywords:** acromioclavicular joint dislocation, coracoid dimensions, Latarjet procedure, tunnel location

## Abstract

**Purpose:**

To assess coracoid process morphology in an Asian population using three‐dimensional (3D) computed tomography (CT) reconstruction and provide reference values for surgical treatment.

**Methods:**

Data on demographic and shoulder CT characteristics were collected from 142 patients for 3D‐CT‐based scapular reconstruction. Ten coracoid morphological indicators and the glenoid width were measured. The morphology of the superior pillar and its undersurface were classified into common shapes. Statistical analyses included intraclass correlation coefficient (ICC) analysis, Cohen's *κ* value, independent samples *t* test, Welch's *t* test, Mann–Whitney *U* test, Kruskal–Wallis test, Spearman and Pearson correlations, receiver operating characteristic (ROC) curves and area under the curve (AUC) values. Relationships among the measured indicators, patient demographics (i.e., sex, age, height and weight) and superior pillar morphology were ascertained.

**Results:**

The intraobserver and interobserver ICC values were 0.924–0.980 and 0.906–0.962, respectively. For intraobserver and interobserver agreement, Cohen's *κ* values were 0.927–0.950 and 0.901–0.937, respectively. Significant sex differences in coracoid measurements were noted. Correlations were observed between the coracoid indicators and glenoid width, sex, height and weight (*p* < 0.05). ROC curve analysis identified height as a significant predictor of safe distance, with cutoff values of 160.5 and 170.5 cm (AUC = 0.82 and 0.83) for women and men, respectively. The superior pillar morphologies included violin (24.65%), long rod (21.13%), short rod (33.80%), trapezoidal (11.97%) and wedge (8.45%) shapes, with the undersurfaces categorized as straight (16.20%), arched (76.76%) and hooked (7.04%), with significant differences in pillar widths among the different morphological types (*p* < 0.05).

**Conclusion:**

Coracoid morphology is crucial in the preoperative planning of given shoulder surgeries, with height and sex serving as key predictors of coracoid graft length. Consideration of variations in the superior pillar shape and undersurface of the coracoid could minimize surgical complications associated with special shoulder surgery.

**Level of Evidence:**

Level IV case series with no comparison.

**Abbreviations**
ANOVA: analysis of variance
AUC: area under the curve
CI: confidence interval
CT: computed tomography
ICC: intraclass correlation coefficient
ROC: receiver operating characteristic
SE: standard error

## INTRODUCTION

The coracoid process is a crucial scapular structure that constitutes a site for the attachment of multiple tendons and ligaments and plays a vital role in shoulder stability [33]. Accurate measurement of the coracoid morphology is particularly important for shoulder surgeries, such as the Latarjet procedure and coracoclavicular ligament reconstruction [6, 43]. Despite the use of various measurement methods, including radiography, computed tomography (CT) and cadaveric examination, in previous studies [18, 19], only limited demographic information, such as sex, age, height and weight, was available in cadaver studies [29]. Owing to the three‐dimensional (3D) complexity of the coracoid process, 3D‐CT imaging‐based data acquisition is recommended for preoperative planning [3, 17]. Regarding the measurement metrics, there is no consensus on the effective coracoid graft length for the Latarjet procedure [43]. Furthermore, data on coracoid morphology in Asian populations are limited, particularly regarding demographic characteristics [12, 19].

In this study, we aimed to perform a detailed 3D‐CT imaging‐based morphometric analysis of the coracoid process to enrich the Asian population‐specific data and explore its application in relevant surgical procedures. We hypothesized that significant correlations exist between coracoid morphology data, glenoid width and patient demographics that could enable an estimation of the effective coracoid graft length during preoperative planning.

## MATERIALS AND METHODS

### Samples

In this study, data on the demographic and radiological characteristics of 142 patients who underwent a CT scan (Brilliance iCT) of the shoulder joint at the Affiliated Hospital of Nanjing University of Chinese Medicine between January 2021 and July 2023 were collected and analyzed. The patients were positioned head‐first in the supine position. The scanning parameters were set as follows: tube voltage of 120 kVp, smart mAs ranging from 50 to 300, a field of view of 350 mm, DoseRight technology, 1‐mm slice thickness, 0.5‐mm slice interval, a pitch of 1 and a rotation speed of 0.75 s per rotation, with two reconstruction series. The study cohort comprised 60 men and 82 women, with 54 left shoulders and 88 right shoulders. The inclusion criteria were as follows: (1) age 18–60 years; (2) no history of trauma, other diseases or surgeries of the ipsilateral shoulder and (3) absence of significant skeletal deformities in the ipsilateral shoulder on CT scans. The exclusion criteria were as follows: (1) a history of scapular instability or anatomical distortion, including fractures, arthritis or skeletal immaturity; (2) indistinct visualization of the coracoid process and (3) congenital deformities of the coracoid process [32].

### Measurements

**Figure 1:**
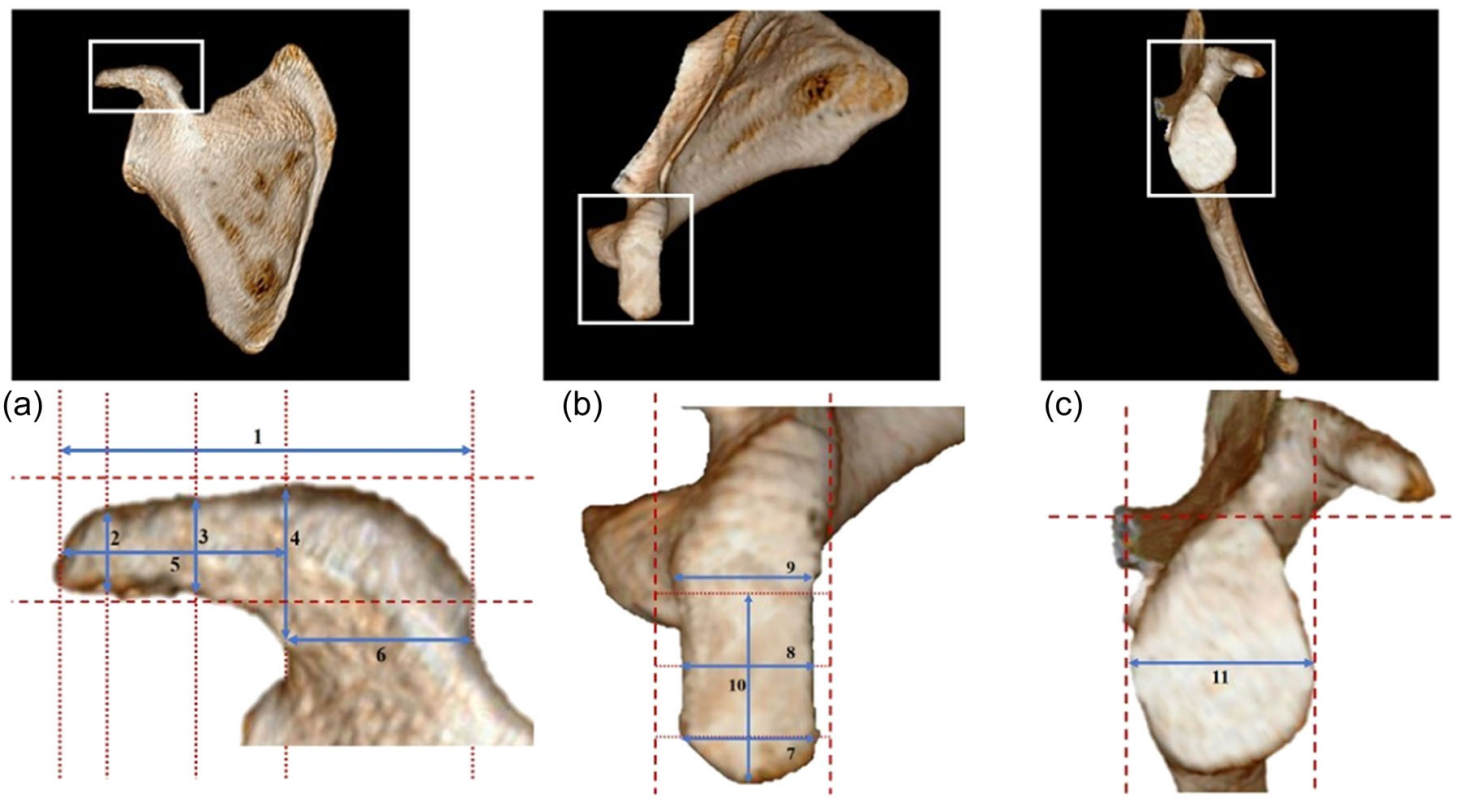
The medial (a), superior (b) and lateral (c) aspects of the coracoid process. The white squares indicate the areas of observation, the blue arrows (1–11) mark the measurement indicators and the dashed red lines present the alignment.

**Figure 2:**
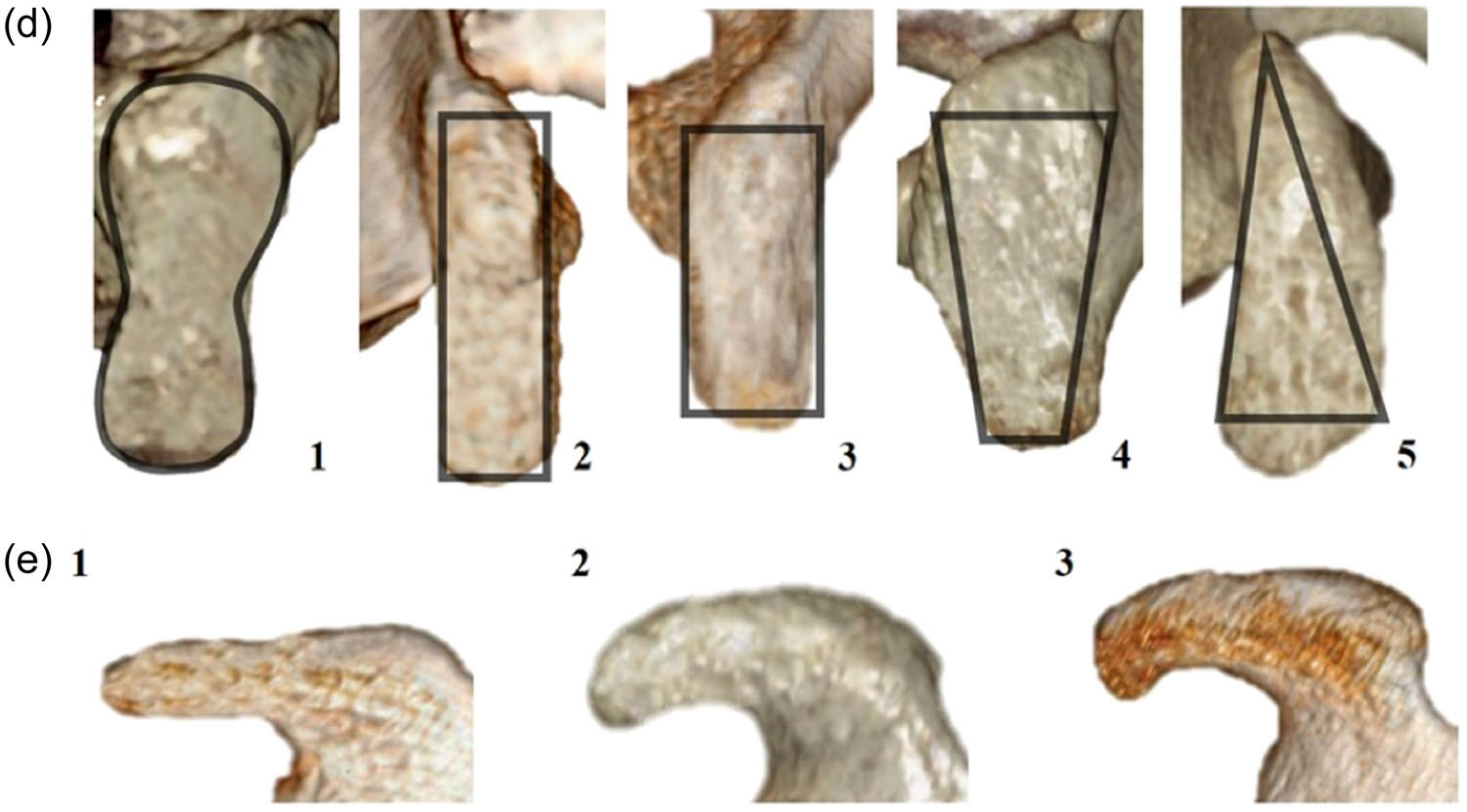
Superior (d) and medial (e) aspects of the coracoid process, with black lines outlining the shapes.

For the 142 participants, 3D scapular reconstruction was performed under the guidance of a radiologist with more than 5 years of experience, and specific measurement sites were identified and analyzed using the Picture Archiving and Communication System measurement tools. We evaluated the anatomical measurements of the coracoid process, the width of the glenoid cavity and superior pillar morphology. Each measurement was performed three times, and the average value of the three measurements was used for analysis. The measurements included the following: (1) coracoid process length (maximum straight‐line distance from the superior pillar tip to the junction where the inferior pillar joins the scapular notch), (2) superior pillar tip thickness (maximum vertical distance at the tip of the superior pillar), (3) superior pillar mid thickness (maximum vertical distance at the midpoint of the superior pillar), (4) superior pillar base thickness (maximum vertical distance at the base of the superior pillar where it joins the reentrant part), (5) superior pillar length (maximum straight‐line distance from the tip to the base of the superior pillar), (6) inferior pillar top width (maximum horizontal distance at the junction of the reentrant part and the inferior pillar), (7) superior pillar tip width (maximum horizontal width at the tip of the superior pillar), (8) superior pillar mid width (maximum horizontal width at the midpoint of the superior pillar), (9) superior pillar base width (maximum horizontal width at the base of the superior pillar where it meets the reentrant part), (10) safety distance (straight‐line distance from the superior pillar tip to a point immediately before the insertion of the coracoclavicular ligament) and (11) width of the glenoid cavity (maximum anteroposterior distance across the glenoid cavity) (Figure 1). Furthermore, the morphology of the superior pillar of the coracoid process was subclassified into five types (Figure 2): (1) violin‐shaped (wide at the tip and base, but narrow in the middle); (2) long rod (narrow superior pillar); (3) short rod (wide superior pillar); (4) trapezoidal (narrow at the tip and wide at the base) and (5) wedge‐shaped (wide at the tip and narrow at the base). Similarly, the shape of the undersurface of the superior pillar was categorized into three types (Figure 2): (1) straight (flat undersurface), (2) arch (curved undersurface) and (3) hooked (bracket‐shaped undersurface).

### Statistical analysis

We assessed differences between the left and right sides as well as sexes and explored correlations with age, height and weight. Receiver operating characteristic (ROC) curve analysis was used to identify factors that influence a safe distance of >20 mm and predict outcomes. Moreover, we compared the coracoid measurements across the five types of superior pillar upper‐surface shapes. Based on the studies by Lian et al. [29] and Jia et al. [19], we estimated a required minimum sample size of 83 participants for our primary parameter, ‘safety distance’, with a margin of error of ±0.5 mm, a 95% confidence level (CI), and 0.80 power.

Interobserver reliability was determined by ensuring that two observers measured all indicators. Intraobserver reliability was assessed by having a single observer conduct measurements twice at a 2‐month interval. Reliability was evaluated using the intraclass correlation coefficient (ICC) and 95% CI with two‐way random‐effects and mixed‐effects models for interobserver and intraobserver agreement, respectively. Cohen's *κ* and a 95% CI were used to assess the reliability of the categorical variables.

Data analysis was performed using SPSS version 27.0. Normality was assessed using the Kolmogorov–Smirnov test. Data with normal distribution were reported as the mean ± SD and analyzed using *t* tests, Welch's *t* test or analysis of variance (ANOVA). Skewed data were reported as median and interquartile range (25th percentile and 75th percentile) and analyzed using the Mann–Whitney *U* or Kruskal–Wallis test. Correlations were evaluated using Pearson's or Spearman's coefficients, as appropriate. ROC curve analysis identified predictors of a safe distance of >20 mm, with separate evaluations for age, height and weight by sex. The cutoff values were determined using the Youden Index. A significance level of α = 0.05 was used, and *p* < 0.05 indicated statistical significance.

## RESULTS

For both intraobserver and interobserver measurements, ICC analysis revealed excellent consistency across all quantitative variables. Calculated using two‐way mixed and random models, the intraobserver and interobserver ICCs of 0.924 to 0.980 and 0.906 to 0.962 indicated high repeatability and strong agreement between observers, respectively. For categorical variables, Cohen's κ values demonstrated substantial to near‐perfect agreement (intraobserver *κ* 0.927–0.950; interobserver *κ* 0.901–0.937) that confirmed the reliability of the classifications.

The measurement of various coracoid process indicators revealed a coracoid process length of 46.62 ± 4.23 mm, superior pillar tip thickness of 9.35 ± 1.38 mm, mid thickness of 10.23 (8.98, 11.53) mm, base thickness of 11.97 ± 1.74 mm and length of 22.19 ± 2.55 mm. The inferior pillar top width was 24.85 (23.16, 26.95) mm, whereas the superior pillar tip width, mid width and base width were 14.11 ± 1.88, 13.87 (12.74, 15.14) and 15.22 (13.94, 16.70) mm, respectively. The safety distance was 21.29 ± 2.37 mm, and the width of the glenoid cavity was 27.39 ± 2.98 mm. The median values of age, height and weight were 46.00 (35.00, 53.00) years, 166.00 (160.00, 171.00) cm and 65.00 (57.00, 75.00) kg, respectively (Table 1). Independent sample *t* tests and the Mann–Whitney *U* test revealed significant sex differences in coracoid measurements, height and weight, but not age (*p *< 0.05; Table 2). Significant differences were observed between the left and right groups in the base width of the superior pillar (*p *< 0.05; Table 3).

**Table 1:** Descriptive statistics of the overall measurements and clinical characteristics (*n* = 142).

Variable(s)	Value
(1) Coracoid process length (mm)	46.62 ± 4.23
(2) Superior pillar tip thickness (mm)	9.35 ± 1.38
(3) Superior pillar mid thickness (mm)	10.23 (8.98, 11.53)
(4) Superior pillar base thickness (mm)	11.97 ± 1.74
(5) Superior pillar length (mm)	22.19 ± 2.55
(6) Inferior pillar top width (mm)	24.85 (23.16, 26.95)
(7) Superior pillar tip width (mm)	14.11 ± 1.88
(8) Superior pillar mid width (mm)	13.87 (12.74, 15.14)
(9) Superior pillar base width (mm)	15.22 (13.94, 16.70)
(10) Safety distance (mm)	21.29 ± 2.37
(11) Glenoid cavity width (mm)	27.39 ± 2.98
Age (years)	46.00 (35.00, 53.00)
Height (cm)	166.00 (160.00, 171.00)
Weight (kg)	65.00 (57.00, 75.00)

**Table 2:** Sex‐stratified comparison of the dimensions of the coracoid process.

Variable(s)	Women (*n* = 82)	Men (*n* = 60)	Statistic	*p* Value
(1) Coracoid process length	44.42 ± 2.87	49.64 ± 3.95	t = −8.69^	<0.01*
(2) Superior pillar tip thickness	8.61 ± 0.92	10.36 ± 1.26	t = −9.13^	<0.01*
(3) Superior pillar mid thickness	9.43 ± 1.26	11.44 ± 1.39	t = −8.96	<0.01*
(4) Superior pillar base thickness	10.77 (10.32, 12.09)	13.19 ± 1.52	Z = −6.85	<0.01*
(5) Superior pillar length	20.75 (19.66, 22.43)	23.67 ± 2.55	Z = −5.63	<0.01*
(6) Inferior pillar top width	24.07 ± 2.11	26.35 ± 2.51	t = −5.89	<0.01*
(7) Superior pillar tip width	13.13 ± 1.38	15.45 ± 1.63	t = −9.15	<0.01*
(8) Superior pillar mid width	13.19 ± 1.49	15.19 ± 1.45	t = −7.99	<0.01*
(9) Superior pillar base width	14.52 ± 1.82	16.46 ± 1.70	t = −6.46	<0.01*
(10) Safety distance	20.19 ± 1.81	22.80 ± 2.23	t = −7.72	<0.01*
(11) Glenoid cavity width	25.74 ± 2.23	29.65 ± 2.32	t = −10.13	<0.01*
Age	44.85 ± 10.17	44.50 (32.25, 53.00)	Z = −0.90	0.37
Height	160.00 (158.00, 165.25)	171.50 (168.25, 178.00)	Z = −8.36	<0.01*
Weight	60.00 (55.00, 65.00)	74.60 (70.00, 81.50)	Z = −7.46	<0.01*

*Note*: Asterisks indicate statistically significant values; ^ indicates Welch's *t* test.

**Table 3:** Comparison of the left and right coracoid processes.

Variable(s)	Left (*n* = 54)	Right (*n* = 88)	Statistic	*p* Value
(1) Coracoid process length	46.48 ± 4.21	45.88 (43.76, 49.20)	Z = −0.15	0.88
(2) Superior pillar tip thickness	8.88 (9.29, 9.85)	9.44 ± 1.37	Z = −1.33	0.18
(3) Superior pillar mid thickness	10.36 ± 1.67	10.23 ± 1.64	t = 0.44	0.66
(4) Superior pillar base thickness	11.97 ± 1.72	11.51 ± 1.75	t = 0.10	0.99
(5) Superior pillar length	22.27 (19.94, 23.97)	21.88 (20.33, 23.86)	Z = −0.06	0.97
(6) Inferior pillar top width	24.84 ± 2.44	25.15 ± 2.61	t = −0.70	0.49
(7) Superior pillar tip width	13.97 ± 2.00	14.20 ± 1.81	t = −0.68	0.50
(8) Superior pillar mid width	13.80 ± 1.76	14.18 ± 1.77	t = −1.24	0.22
(9) Superior pillar base width	14.81 ± 1.80	15.66 ± 2.07	t = −2.49	0.01*
(10) Safety distance	21.18 (19.28, 23.01)	21.35 ± 2.42	Z = −0.29	0.77
(11) Glenoid cavity width	26.78 (24.20, 29.51)	27.69 ± 2.95	Z = −1.37	0.17

*Note*: Asterisks indicate statistically significant values.

Spearman and Pearson correlation analyses showed that the coracoid indicators correlated with the width of the glenoid cavity, sex, height and weight (*p* < 0.05). Age correlated only with the base thickness of the superior pillar and width of the glenoid cavity (*p *< 0.05, Table 4). In women, all coracoid measurements, except base thickness, correlated with the width of the glenoid cavity (*p* < 0.05). The superior pillar width, safe distance and width of the glenoid cavity correlated with age (*p* < 0.05). All measurements, except for the tip and base thicknesses of the superior pillar, correlated with height (*p *< 0.05, Table 5). In men, the length of the coracoid process correlated with the width of the glenoid cavity and weight (*p* < 0.05). The superior pillar tip thickness, mid thickness and safety distance correlated with the width of the glenoid cavity, height and weight (*p* < 0.05). Superior pillar base thickness correlated with weight (*p* < 0.05), whereas the superior pillar length correlated with both height and weight (*p* < 0.05). The superior pillar tip width correlated with the width of the glenoid cavity and height (*p* < 0.05), whereas the mid width correlated with height (*p* < 0.05). The width of the glenoid cavity correlated with age (*p *< 0.05, Table 6).

**Table 4:** Correlation analysis of the overall measurements of the coracoid process.

Variable(s)	(11) Glenoid cavity width		Gender		Age		Height		Weight	
	*r*	*p* Value	*r*	*p* Value	*r*	*p* Value	*r*	*p* Value	*r*	*p* Value
(1) Coracoid process length	0.64 ^a^	<0.01*	0.61	<0.01*	0.07	0.37	0.64	<0.01*	0.48	<0.01*
(2) Superior pillar tip thickness	0.60 ^a^	<0.01*	0.64	<0.01*	−0.77	0.36	0.54	<0.01*	0.55	<0.01*
(3) Superior pillar mid thickness	0.56	<0.01*	0.60	<0.01*	−0.34	0.69	0.62	<0.01*	0.52	<0.01*
(4) Superior pillar base thickness	0.43 ^a^	<0.01*	0.58	<0.01*	−0.18	0.03*	0.50	<0.01*	0.48	<0.01*
(5) Superior pillar length	0.47 ^a^	<0.01*	0.47	<0.01*	0.15	0.07	0.60	<0.01*	0.35	<0.01*
(6) Inferior pillar top width	0.51	<0.01*	0.43	<0.01*	−0.04	0.62	0.40	<0.01*	0.37	<0.01*
(7) Superior pillar tip width	0.62 ^a^	<0.01*	0.61	<0.01*	0.13	0.13	0.60	<0.01*	0.41	<0.01*
(8) Superior pillar mid width	0.62	<0.01*	0.58	<0.01*	0.13	0.13	0.56	<0.01*	0.39	<0.01*
(9) Superior pillar base width	0.55	<0.01*	0.51	<0.01*	0.01	0.87	0.46	<0.01*	0.39	<0.01*
(10) Safety distance	0.55 ^a^	<0.01*	0.53	<0.01*	0.09	0.27	0.70	<0.01*	0.48	<0.01*
(11) Glenoid cavity width			0.67	<0.01*	0.22	0.01*	0.60	<0.01*	0.50	<0.01*

*Note*: Asterisks indicate statistically significant values; a, Pearson's correlation coefficient; *r*, correlation coefficient.

**Table 5:** Correlation analysis of coracoid measurements in women (*n* = 82).

Variable(s)	(11) Glenoid cavity width		Age		Height		Weight	
	***r***	***p*** **Value**	***r***	***p*** **Value**	***r***	***p*** **Value**	***r***	***p*** **Value**
(1) Coracoid process length	0.52 ^a^	<0.01*	0.20 ^a^	0.08	0.44	<0.01*	−0.00	0.98
(2) Superior pillar tip thickness	0.27 ^a^	0.01*	−0.01 ^a^	0.95	0.13	0.25	0.18	0.11
(3) Superior pillar mid thickness	0.26^a^	0.02*	0.03 ^a^	0.77	0.33	<0.01*	0.18	0.10
(4) Superior pillar base thickness	0.10	0.36	−0.15	0.17	0.18	0.10	0.17	0.14
(5) Superior pillar length	0.24	0.03*	0.16	0.16	0.43	<0.01*	0.02	0.87
(6) Inferior pillar top width	0.48 ^a^	<0.01*	0.03 ^a^	0.80	0.22	0.05*	0.01	0.93
(7) Superior pillar tip width	0.38 ^a^	<0.01*	0.18 ^a^	0.11	0.26	0.02*	−0.03	0.80
(8) Superior pillar mid width	0.45 ^a^	<0.01*	0.24 ^a^	0.03*	0.36	<0.01*	0.12	0.30
(9) Superior pillar base width	0.38 ^a^	<0.01*	0.06 ^a^	0.60	0.24	0.03*	0.00	1.00
(10) Safety distance	0.34 ^a^	<0.01*	0.22 ^a^	0.05*	0.60	<0.01*	0.19	0.08
(11) Glenoid cavity width			0.25 ^a^	0.02*	0.32	<0.01*	0.10	0.63

*Note*: Asterisks indicate statistically significant values; a, Pearson's correlation coefficient; *r*, correlation coefficient.

**Table 6:** Correlation analysis of coracoid measurements in men (*n* = 60).

Variable(s)	(11) Glenoid cavity width		Age		Height		Weight	
	*r*	*p* Value	*r*	*p* Value	*r*	*p* Value	*r*	*p* Value
(1) Coracoid process length	0.30 ^a^	0.02*	0.14	0.27	0.19	0.15	0.32	0.02*
(2) Superior pillar tip thickness	0.39 ^a^	<0.01*	−0.02	0.86	0.27	0.04*	0.40	<0.01*
(3) Superior pillar mid thickness	0.30 ^a^	0.02*	0.02	0.88	0.37	<0.01*	0.39	<0.01*
(4) Superior pillar base thickness	0.03 ^a^	0.80	−0.12	0.34	0.08	0.57	0.26	0.04*
(5) Superior pillar length	0.25 ^a^	0.06	0.24	0.07	0.31	0.02*	0.26	0.05*
(6) Inferior pillar top width	0.20 ^a^	0.12	0.02	0.91	−0.02	0.89	0.21	0.11
(7) Superior pillar tip width	0.36 ^a^	<0.01*	0.19	0.15	0.43	<0.01*	0.18	0.18
(8) Superior pillar mid width	0.22 ^a^	0.09	0.14	0.28	0.26	0.05*	−0.00	0.99
(9) Superior pillar base width	0.25 ^a^	0.05	0.12	0.37	0.09	0.52	0.19	0.16
(10) Safety distance	0.28 ^a^	0.03*	0.14	0.28	0.42	<0.01*	0.34	0.01*
(11) Glenoid cavity width			0.52	<0.01*	0.02	0.88	0.09	0.48

*Note*: Asterisks indicate statistically significant values; a, Pearson's correlation coefficient; *r*, correlation coefficient.

**Figure 3:**
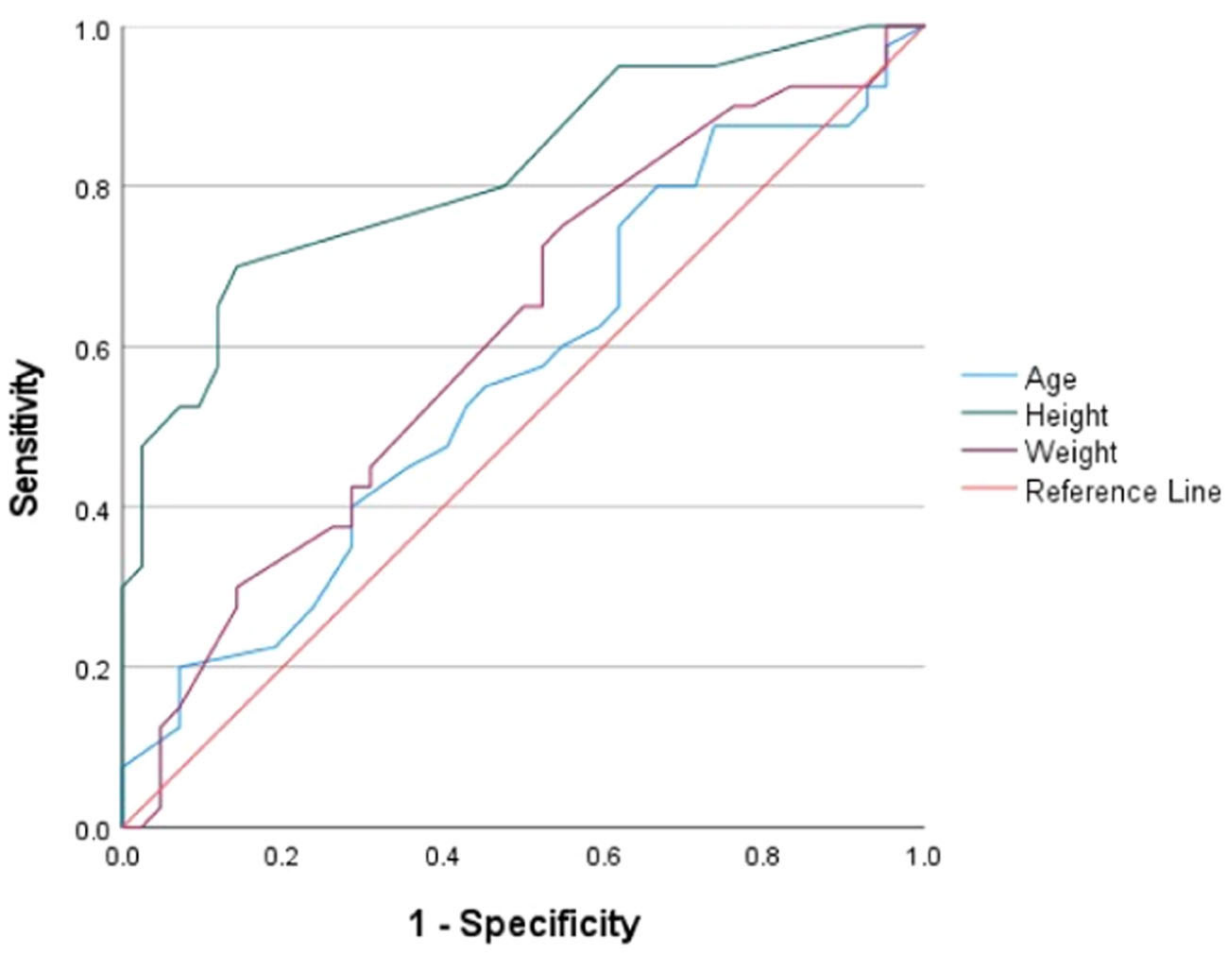
Receiver operating characteristics curves for predicting the safety distance of the coracoid process in women.

**Figure 4:**
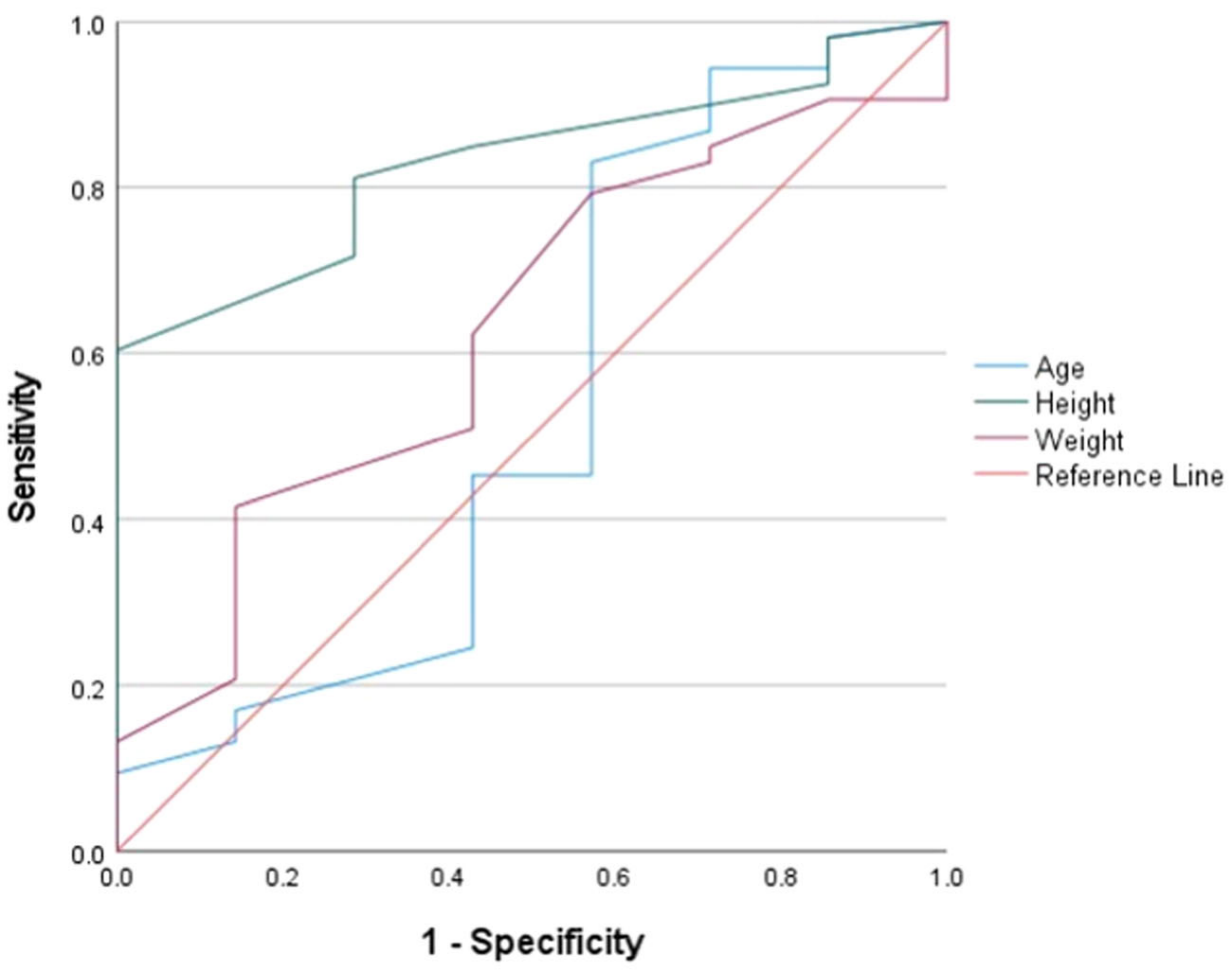
Receiver operating characteristics curves for predicting the safety distance of the coracoid process in men.

Height and weight were identified as influencing factors and used to establish ROC curve models for women (Figure 3) and men (Figure 4). In women and men, height was a significant predictor of safe distance (area under the curve = 0.82 and 0.83) with cutoff values of 160.5 and 170.5 cm, respectively (Table 7).

**Table 7:** Analysis of the receiver operating characteristics curves.

Gender	Variable(s)	AUC	SE	*p* Value	Cutoff point (Youden index)	95% CI
Women	Age	0.57	0.06	0.31	35.50 (0.14)	0.44 ~ 0.69
	Height	0.82	0.05	<0.01*	160.50 (0.56)	0.73 ~ 0.91
	Weight	0.62	0.06	0.07	55.50 (0.20)	0.49 ~ 0.74
Men	Age	0.54	0.14	0.75	30.50 (0.26)	0.27 ~ 0.81
	Height	0.83	0.06	<0.01*	170.50 (0.60)	0.71 ~ 0.95
	Weight	0.63	0.11	0.27	75.50 (0.27)	0.42 ~ 0.84

*Note*: Asterisks indicate statistically significant values.

Abbreviations: AUC, area under the curve; CI, confidence interval; SE, standard error.

The superior pillar morphology of the coracoid process was violin‐shaped in 35, long rods in 30, short rods in 48, trapezoidal in 17 and wedge‐shaped in 12 cases. The impact of these five morphologies on the coracoid measurements was compared using ANOVA or the Kruskal–Wallis test and revealed significant differences in superior pillar tip width, mid width and base width (*p *< 0.05; Table 8). Morphological classification of the superior pillar undersurface included 23 straight, 109 arched and 10 hooked cases.

**Table 8:** Comparison of the morphological dimensions of the superior pillar of the coracoid process.

Variable	Violin‐shaped (*n* = 35)	Long rod (*n* = 30)	Short rod (*n* = 48)	Trapezoidal (*n* = 17)	Wedge‐shaped (*n* = 12)	Statistic	*p* Value
(1) Coracoid process length	45.19 (42.37, 49.53)	46.31 ± 3.94	47.08 (44.66, 49.40)	46.84 (42.95, 49.57)	47.45 (45.13, 48.09)	H = 2.88#	0.58
(2) Superior pillar tip thickness	9.58 (8.31, 10.25)	8.97 ± 1.35	9.63 (8.68, 10.59)	9.04 (7.94, 9.54)	9.12 (8.27, 10.35)	H = 7.86#	0.10
(3) Superior pillar mid thickness	10.44 ± 1.83	10.23 ± 1.54	10.46 ± 1.65	9.66 ± 1.58	10.19 ± 1.42	F = 0.83	0.51
(4) Superior pillar base thickness	12.25 ± 1.74	11.86 ± 1.72	12.09 ± 1.88	10.80 (10.47, 11.99)	11.89 ± 1.60	F = 0.87	0.49
(5) Superior pillar length	22.09 ± 2.82	22.62 ± 2.65	22.13 ± 2.49	21.45 ± 2.32	22.72 ± 2.03	F = 0.72	0.58
(7) Superior pillar tip width	14.85 ± 2.12	13.15 ± 1.50	14.08 ± 1.51	13.75 ± 1.87	14.99 ± 2.31	H = 13.56	<0.01*
(8) Superior pillar mid width	13.78 ± 1.96	13.12 ± 1.51	14.36 ± 1.49	14.60 ± 1.85	14.96 ± 1.84	F = 4.18	<0.01*
(9) Superior pillar base width	15.31 ± 2.05	14.17 ± 1.97	15.65 ± 1.63	15.84 ± 1.83	16.38 ± 2.58	F = 4.22	<0.01*
(10) Safety distance	21.56 ± 2.68	21.58 ± 2.39	21.09 ± 2.28	20.21 ± 1.82	22.11 ± 2.17	F = 1.58	0.18

*Note*: Asterisks indicate statistically significant values; #, indicates the Kruskal–Wallis test.

## DISCUSSION

The Latarjet procedure, first introduced by Latarjet in 1954, remains the cornerstone of treatment for recurrent anterior shoulder instability [26] and involves the harvesting of a coracoid process graft to fill the glenoid bone defect, and is particularly reliable in cases with glenoid bone loss >25% and more dependable than Bankart repair [5, 15]. Lafosse et al. [25] suggested a bone graft length of 20–25 mm, whereas Walch et al. [35] recommended a coracoid graft length >25 mm to enable safe accommodation of two 4.5‐mm screws. Frank et al. [13] proposed a minimum coracoid harvest length of 20 mm. Latarjet [26] underscored the protection of the coracoclavicular ligaments at the osteotomy site and defined the ‘safe distance’ as the length from the tip of the coracoid process to the anterior margin of the insertion of the coracoclavicular ligaments. Nonetheless, the reported safe distances vary considerably according to Dolan et al. [9] (28.50 mm), Shibata et al. [39] (24.8 ± 3.4 mm), Lian et al. [29] (23.93 mm), Bhatia et al. [4] (19.00 mm) and Jia et al. [19] (20.80 ± 2.02 mm). In our study, we found a safe distance of 21.29 ± 2.37 mm in the Asian population, and for women, the safe distance was 20.19 ± 1.81 mm, which is less than the Walch et al. recommended 25‐mm ideal length. Notably, 49 patients had a safe distance of <20 mm, with the lowest distance of 15.63 mm in women. This discrepancy complicates the standard Latarjet procedure. Compared with the safe distance length of the coracoid process reported by Dolan et al. [9] in Western populations, the safe distance measured in this study was noticeably shorter. Jiang et al. [20] observed shorter coracoid lengths in Asians that are frequently inadequate for securing two 4.5‐mm screws. Similarly, Lian et al. [29] reported a reduced safe distance in Chinese individuals that sometimes allowed only one 4.5‐mm screw insertion [28]. Boutsiadis et al. [6], adhering to AO principles, posited that the minimum safe distance between the coracoid graft's screw head and the osteotomy line should at least equal to the screw's diameter and proposed that using 3.5‐mm screws instead of 4.5‐mm screws can increase the proportion (from 56% to 87%) of cases with a safe distance. Based on these findings, our study supports the notion that, in Asian patients who undergo the Latarjet procedure and have a coracoid process length that may be insufficient for the traditional approach, the use of smaller implants, such as 3.5‐mm screws, can provide sufficient safety margins to reduce the risk of iatrogenic injury.

Evaluation of the thickness of the superior pillar of the coracoid process is crucial for determining the size of the glenoid cavity defect that needs to be filled. In this study, we measured the maximum anteroposterior distance of the glenoid cavity, which is consistently located in the middle to lower regions of the cavity [1, 17]. The average thicknesses of the superior pillars at the tip, midsection and base were 34.22%, 37.62% and 43.93% of the width of the glenoid cavity, respectively. The minimum thickness percentages at these locations were 26.17%, 27.11% and 32.69%, respectively, which is consistent with the data from the extant research [34]. Posey et al. [36] observed that, in 20% of the cases, the coracoid graft thickness was insufficient for defects of up to 30% of the glenoid, with which our finding that 17% of the samples could not fill a 30% defect is aligned [23, 28]. Our study found that the widths of the superior pillars at the tip, middle and base were consistently greater than their corresponding thicknesses, which is consistent with the data in the published research [6, 36]. Therefore, for patients with glenoid defects ≥30%, consideration of modified Latarjet or Eden–Hybinette techniques is advised [6, 31]. Additionally, the application of Latarjet techniques should consider anatomical requirements, patient preferences and functional demands. The ISIS score provides recommendations for treatment selection, with scores >6 favoring Latarjet surgery [2, 30]. Height, particularly when analyzed separately for each sex, showed a broader correlation with multiple measured indices, aligning with the conclusions drawn by Jia et al. [19], Shibata et al. [39] and Posey et al. [36]. The selective correlation of age with some metrics may be attributed to the limited growth potential of the coracoid tip, which ceases around age 25 [14]. Posey et al. [36] used quantile regression modelling to suggest that 90% of children and adolescents with height ≥163 cm have a coracoid length of at least 25 mm. Our study's ROC curve indicates that, in women and men taller than 160.5 and 170.5 cm, respectively, it is more likely to obtain at least 20‐mm long coracoid grafts.

The Rockwood classification categorizes acromioclavicular joint injuries into types I–VI, wherein types IV–VI require surgical intervention [40]. The Triple Endobutton procedure for Rockwood III–V dislocations outperforms other methods in pain reduction, shoulder mobility, biomechanical strength and stability [21, 22, 27, 37, 42]. The triple endobutton procedure is ideal for acromioclavicular dislocations, although its effectiveness depends on the coracoid bone tunnel dimensions and positioning [41]. Notably, tendon grafts that require larger tunnels may potentially increase the risk of fractures [10]. Coale et al. [8] documented the occurrence of medial cortical fractures following clavicle–coracoid drilling and suggested that isolated coracoid tunnel drilling is more suitable when considering the anatomical structures and biomechanical integrity. Kummer et al. [24] found that a 6‐mm hole at the coracoid base offers higher strength and lower fracture risk than lateral drilling, which supports Rylander's finding that larger holes weaken bone strength [38]. Campbell et al. [7] found that, compared to 6‐mm tunnels, 4.5‐mm tunnels could bear higher loads. In our study, the coracoid's superior pillar base width was 15.22 (13.94–16.70) mm, which was <15 mm in 64 cases, and the smallest width was 11.30 mm. This suggests that a top–down drilled tunnel with a diameter <4 mm at the superior pillar base is safer when considering the anatomical characteristics of the Asian population.

In this study, the superior pillar of the coracoid process was classified into five morphologies, with the short rod shape being the most common (33.80%). Zhang et al. [43] identified five shapes in the Chinese population: figure‐eight, long stick, short stick, teardrop and wedge, wherein the figure‐eight and short stick shapes were predominant. Guo et al. [16] indicated that peanut, short rod and melon seed shapes at the base offer broader widths, and thereby reduce fracture risks. In our study, the violin (24.65%) and trapezoidal (11.97%) shapes narrowed from the base to the middle, which suggested an increased risk of cortical rupture with anterior tunnel placement in the Endobutton procedure for acromioclavicular joint dislocation. Hardy et al. [17] described the coracoid's inferior surface shapes as flat (16.67%), curved (66.67%) or banana‐shaped (8.33%), wherein banana shapes resulted in a potential mismatch with the anterior edge of the glenoid. Our observations identified straight (16.20%), arc (76.76%) and hook (7.04%) inferior surface shapes, with the arc and hook shapes potentially causing impingement and tendon damage [11].

This study has some limitations, including a restricted sample size for coracoid measurements, which necessitates further large‐scale research to refine the measurement data and statistical outcomes. Coracoid morphology exhibits specificity, and 3D‐CT reconstruction has some constraints, such as pectoralis minor‐related variations that affect graft length and unclear coracoclavicular ligament endpoints in some samples. Additionally, the measurement process did not account for the angular factors of the coracoid morphology, potentially leading to discrepancies between the measured values and the actual conditions.

In conclusion, measurements of the coracoid morphology provide crucial clinical guidance for shoulder surgeries. The shape and size of the coracoid vary across populations, and Asian patients typically have smaller dimensions than Western patients. Preoperative considerations should include the length of the coracoid graft, the size of the internal fixation implants, and the dimensions for drilling the coracoid bone tunnels. Furthermore, correlations among height, sex and coracoid measurement indices can serve as preoperative indicators for estimating the coracoid graft length.

## AUTHOR CONTRIBUTIONS

Luning Sun designed the study. Su Yan and Hao Shu performed the literature review, created the figures and tables and conducted the data analysis. Tongyue Ji and Chao Lu measured the clinical parameters from patients' CT scans. Luning Sun and Tongyue Ji edited and polished the manuscript. All authors have read and approved the final manuscript submitted for publication.

## CONFLICT OF INTEREST STATEMENT

The authors declare no conflicts of interest.

## ETHICS STATEMENT

This study is a retrospective observational analysis. The Research Ethics Committee of the Affiliated Hospital of Nanjing University of Chinese Medicine has determined that ethical approval is not necessary for this research. Consequently, given the study's noninterventional design and the use of anonymized data, informed consent was not required.

## Data Availability

Data are available from the corresponding author.
